# A system of vectors for *Bacillus subtilis* spore surface display

**DOI:** 10.1186/1475-2859-13-30

**Published:** 2014-02-24

**Authors:** Adam Iwanicki, Iwona Piątek, Małgorzata Stasiłojć, Anna Grela, Tomasz Łęga, Michał Obuchowski, Krzysztof Hinc

**Affiliations:** 1 Department of Medical Biotechnology, Intercollegiate Faculty of Biotechnology UG-MUG, Medical University of Gdańsk, Dębinki 1, Gdańsk 80-211, Poland

**Keywords:** *Bacillus subtilis*, Spore display, Ectopic integration vectors

## Abstract

**Background:**

Bacterial spores have been utilized as platforms for protein display. The best studied display systems are based on *Bacillus subtilis* spores in which several coat proteins have successfully been used as anchors for heterologous protein. Increasing knowledge about spore coat structure enables selection of new anchor proteins such as CotZ and CgeA. Here we describe a system of vectors for display of proteins on the surface of *B. subtilis* spores.

**Results:**

We have designed and constructed a set of 16 vectors for ectopic integration which can be used for spore surface display of heterologous proteins. There is a selection of five coat proteins: CotB, CotC, CotG, CotZ and CgeA which can be used for construction of fusions. Three of these (CotB, CotC and CotG) enable obtaining N-terminal and C-terminal fusions and other two (CotZ and CgeA) are designed to produce C-terminal fusions only. All the vectors enable introduction of an additional peptide linker between anchor and displayed protein to enhance surface display. As a selection marker trophic genes are used. Additionally we describe an example application of presented vector system to display CagA protein of *Helicobacter pylori* in fusion with CgeA spore coat protein.

**Conclusions:**

Described system of vectors is a versatile tool for display of heterologous proteins on the surface of *B. subtilis* spores. Such recombinant spores can be further used as for example biocatalysts or antigen-carriers in vaccine formulations. The lack of antibiotic resistance genes in the system makes such spores an interesting option for applications in which a possible release to the environment can occur.

## Background

The targeting and anchoring of heterologous proteins and peptides to the outer surface of bacteriophages and cells is becoming a tool for research in the fields of microbiology, molecular biology or vaccinology, as well as is utilized in the biotechnological industry. This approach is based on the idea of using naturally occurring surface proteins as an anchor for targeting proteins of interest, also called passenger proteins. Such recombinant phages or cells can be used in many applications, to mention only a few examples, such as vaccine vehicles, carriers of active enzymes and antibodies, biosensors (for review see [1-4]). The main problem concerning usage of these systems is connected with the need of the chimeric protein to cross the cytoplasmic membrane. Not every chimera is able cross this barrier resulting in failure of surface display. Most of these limitations can be overcome by using bacterial endospores. Classic examples of spore forming microorganisms are bacteria from genus *Bacillus*. Formation of endospores is a strategy used by these bacteria to survive unfavorable and growth-restricting conditions. *Bacillus* is well known aerobic bacterium widely used for production of industrial proteins. It is classified as a GRAS organism (generally recognized as safe), possesses low trophic requirements and serves as a model Gram-positive microorganism. One of the main advantages of *Bacillus* spores as vehicles for surface display of proteins is that these structures are formed inside of mother cell in natural process of sporulation. Additionally, a set of molecular chaperones active in the cytoplasm of bacteria can facilitate proper folding of the heterologous protein.

The process of sporulation is controlled by a multistep developmental program starting with formation of polar septum. Upon formation of asymmetric compartments the mother cell initiates the engulfment of the forespore, which upon completion of this process becomes double-membrane bound structure. As the engulfment proceeds two external protective layers are built: the cortex, composed of peptidoglycan [5], assembled between the inner and outer forespore membranes, and the proteinaceous coat, the outermost spore layer [6,7]. The central part of the spore is the core, containing partially dehydrated cytoplasm.

The spore coat is composed of at least 70 individual proteins, which are produced in the mother cell [8] and consists of three distinct layers: a lamellar inner coat, a more coarsely layered outer coat and the crust [9,10]. While five proteins are crucial for proper spore coat formation (SpoIVA, SpoVM, SpoVID, SafA and CotE) and therefore they are called morphogenetic proteins [11], another three proteins, CotX, CotY and CotZ, have been shown to be morphogenes of the crust [11,12].

So far four spore coat proteins have been used for display of enzymes or antigens in spore based vaccines. These are CotB, CotC, CotG and CotX. All of them are located in the outermost layer of the coat. CotB protein has been used for display of C-terminal part of *Clostridium tetani* toxin [13], 1b-3 and 4 domains of the Protective Antigen of *Bacillus anthracis*[14] and subunit A of *Helicobacter acinonychis* urease [15]. This last one has also been displayed using CotC and CotG proteins. CotC served as an anchor for such proteins as teugmental protein of *Clonorchis sinensis*[14] or human serum albumin [16]. CotG protein has been used for display of GFP [17] or heterologous enzymes [18,19]. Recently, a crust protein CotZ has been shown to be a good candidate as a new anchor protein useful in spore surface display [20].

Here we present a system of 16 integration vectors which can be used for display of heterologous proteins on the spore surface utilizing CotB, CotC, CotG, CotZ or CgeA spore coat proteins. Vectors are prepared in the way enabling for cloning a gene of interest to at the N- or C-terminus of a gene coding for spore coat protein with an optional peptide linker. Each vector harbors proper trophic gene, which ensures genetic stability and selection for appropriate recombinant strains. Lack of antibiotic resistance genes makes the use of such vector system safe in case of applications in which prepared recombinant spores could enter the environment. Such system could be easily used for preparation of spore-based vaccines or as biocatalysts.

## Results and discussion

### The design of pCot vectors

The prototype vector of the pCot set has the following properties. (i) It cannot replicate in *B. subtilis* but carries ColE1 replication sequences and a β-lactamase gene for amplification in *E. coli*. (ii) The *cot*/*cgeA* gene is cloned along with its original promoter to ensure proper expression of obtained fusion protein. Depending on the designation of the vector a cloning site is introduced either at the N- or C-terminus of the *cot* gene. (iii) Genetic stability and selection of recombinant strains is possible due to presence of selection marker – a trophic gene cassette enabling for complementation in recipient auxotrophic strain. It is worth stressing out that no antibiotic resistance gene selectable in *B. subtilis* is introduced into the vectors. Such design of the system is especially important for applications in which recombinant spores can be released to the environment. In that case it prevents from shedding the antibiotic resistant bacteria. A trophic gene cassette is cloned downstream of the *cot* gene to prevent transcription interference. (iv) The *cot*/*cgeA* gene along with trophic gene cassette is flanked by fragments of non-essential gene to enable ectopic integration into the chromosome of recipient strain.

Five spore coat proteins have been selected as carriers of heterologous proteins. The usefulness of four proteins CotB, CotC, CotG and CotZ for such application has already been shown. Additionally a crust protein CgeA has also been selected.

In case of well described CotBCG proteins we decided to prepare four variants of the vectors. Two enable producing recombinant protein fused to C-terminus of Cot protein while the other two are designed for N-terminal fusions. Each pair consists of one version of vector in which recombinant protein is linked directly to Cot protein and the other with short peptide linker (-GGGGS-). The vectors with *cotZ* and *cgeA* genes are designed in a way enabling for obtaining fusions with heterologous protein attached to the C-terminus of the coat protein directly or via alpha-helical linker (-GGGEAAAKGGG-) [21]. Such set of vectors gives a flexibility in construction of strains producing recombinant spores. The efficiency of expression and surface display of heterologous protein depends on its nature. It is known, that in case of some protein fusions direct link between two partners may result in fusion instability or aggregation [22,23]. In case of spore surface display such situation would prevent coat protein-passenger protein fusion from incorporation into the spore coat structure. Linking of heterologous protein to the coat protein by a linker might help to solve these problems. A choice of type of fusion with spore coat protein gives an opportunity to optimize the display.

Vectors for C-terminal fusions harbor a *cot*/*cgeA* gene cloned along with its original promoter and ribosome binding site (RBS). A *cot*/*cgeA* gene has no STOP codon but a multiple cloning site is located right at the end of the gene or just after the sequence coding for a peptide linker. It is important to clone a gene of interest along with its own STOP codon to ensure proper termination of translation.

Vectors for N-terminal fusions have a multiple cloning site inserted in between START codon and the remaining part of a *cot* gene. In variants with linkers a linker coding sequence is inserted directly downstream of the multiple cloning site. In case of N-terminal fusion vectors it is important to verify, that a gene of interest is cloned in frame of the *cot* gene translation and it has no STOP codon.

Ectopic integration loci are selected as follows: pCotB, pCotZ and pCgeA vectors – *amyE*, pCotC vectors – *lacA* and pCotG vectors – *pyrD*. All the loci are non-essential for growth of *B. subtilis* in normal conditions and are commonly used for introducing cloned genes or constructs into the chromosome of this bacterium. The use of different integration loci makes the system more flexible and enables designing a recombinant strains displaying on the spore surface three different fusions at the same time.

**Figure 1:**
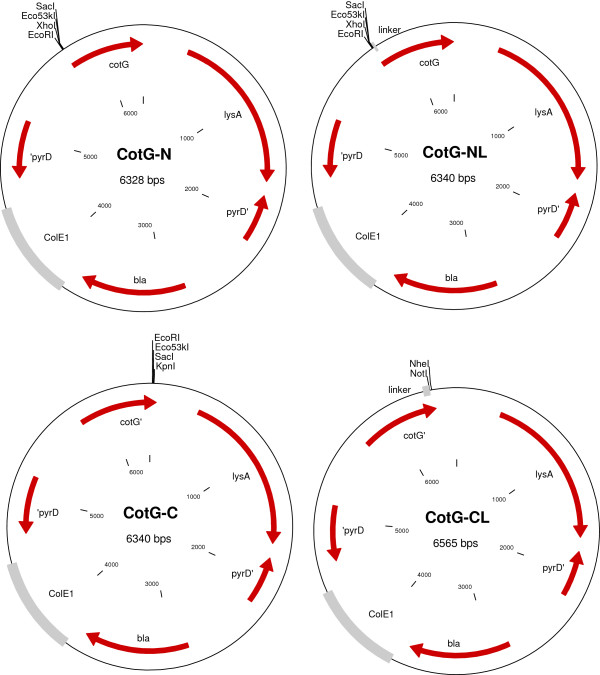
**Schematic maps of the *cotG *vector series.** Indicated sites for restriction enzymes are available for cloning of a gene encoding a protein to be displayed on the spore surface.

**Figure 2:**
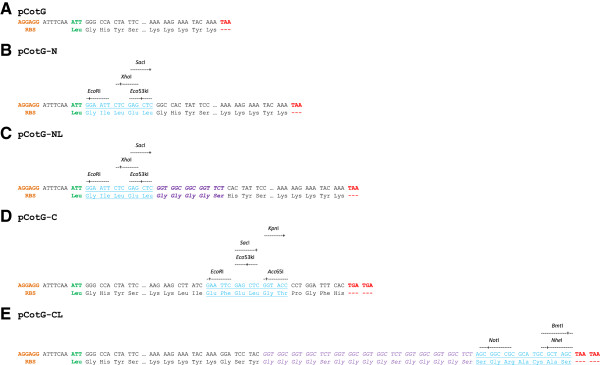
**The *cotG *vector series. A** – 5’ and 3’ end of *cotG* gene. **B** – multicloning site present in the pCotG-N vector. **C** – multicloning site and peptide linker present in pCotG-NL vector. **D** – multicloning site present in pCotG-C vector. **E** – peptide linker and multicloning site present in pCotG-CL vector. Colour labels: orange – ribosome binding site of *cotG*, green – start codon, blue – multicloning site, violet – peptide linker, red – stop codon.

The following genes are used as selection markers in the vector system: pCotB – *thrC*, pCotC, pCotZ, pCgeA – *trpC* and pCotG – *lysA*. Recipient strains compatible with these vectors should be the auxotrophs for appropriate amino acids. The auxotrophy can be achieved by inactivation, deletion or complete knockout [24] of the gene used for selection. Selection of recombinant strains upon transformation can then be done using plates with minimal medium lacking threonine, tryptophan or lysine. As an example vectors of pCotG series are shown in Figures 1 and 2.

### Construction of pCgeA-CagA vectors

**Figure 3:**
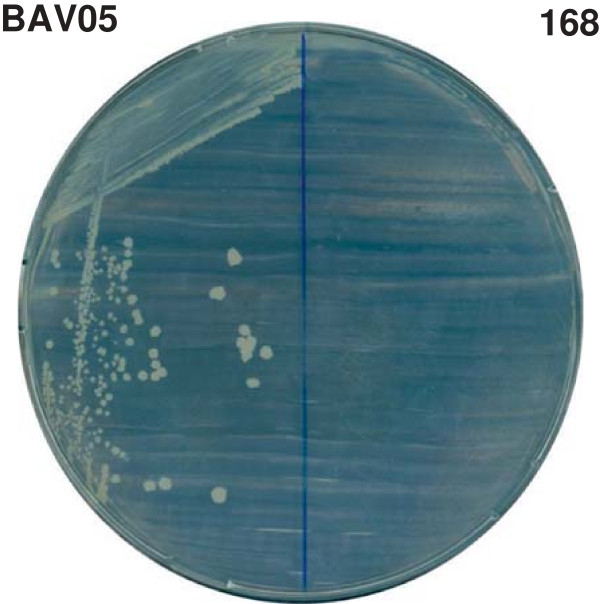
Growth of recombinant strain BAV05 and wild-type strain 168 on minimal plates lacking tryptophan.

To test the system we have prepared two vectors for display of a fragment of *Helicobacter pylori* CagA protein (cytotoxic-associated gene A) in fusion with C-terminal end of CgeA protein. The heterologous part of the fusion encompassed 83 amino acids (residues 1146 to 1222) of CagA and was selected from the most immunogenic regions of this protein as designated by Antigen program (a part of EMBOSS package; http://emboss.sourceforge.net/). One vector enabled production of direct fusion of CgeA and CagA fragment, while in the other the heterologous part was connected via alpha-helical linker. Obtained plasmid were named pAV05 (*cgeA*-*cagA*) and pAV03 (*cgeA*-linker-*cagA*) and used for transformation of *Bacillus subtilis* strain 168. Laboratory strain 168 is an auxotroph for tryptophan due to mutation in *trpC* gene. The presence of functional *trpC* gene (cloned from *B. subtilis* 3610) within the region, which is integrated into the *amyE* locus along with *cgeA*-*cagA* fusion upon successful recombination enabled us to select prototrophic recombinant strains (Figure 3).

Both strains and their isogenic parental strain 168 showed comparable sporulation and germination efficiencies and their spores were equally resistant to chloroform and lysozyme treatment (not shown). Therefore, limited to the spore properties that we have analyzed, the presence of CgeA-CagA and CgeA-linker-CagA fusions did not affect spore structure or functionality.

### Spore coat expression

**Figure 4:**
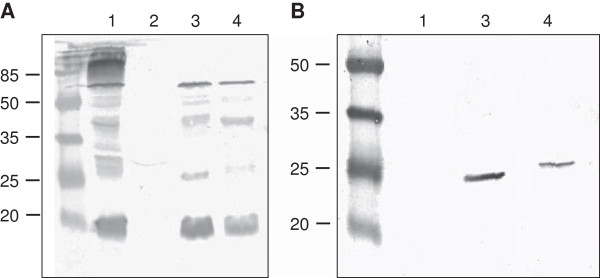
**Western blotting analysis of expression of the *cgeA-cagA *fusion gene.** Spore coat proteins were extracted and analysed by western blotting with anti-CgeA **(panel A)** or anti-CagA **(panel B)** antibodies. Spore coat proteins from spores of the 168 (lane 1), the BAV08 (inactivation of *cgeA*) (lane 2), the BAV05 (fusion CgeA-CagA) (lane 3) and the BAV07 (fusion CgeA-linker-CagA) (lane 4) strains. Each lane of panel **A** and **B** was loaded with 20 μg of total proteins.

The localization of fusion proteins on the spore coat was tested by western blotting with anti-CgeA and anti-CagA antibodies. The analysis of spore coat proteins purified from the wild type and recombinant strains BAV05 carrying fusion CgeA-CagA revealed the presence of an about 23-kDa band, which reacted with both CgeA- and CagA-specific antibodies (Figure 4). In case of strain BAV07 (CgeA-linker-CagA) we observed an about 24-kDa band, which also reacted with both types of antibodies (Figure 4). Calculated molecular masses of fusion proteins are 22.8 kDa for CgeA-CagA and 23.6 kDa for fusion with linker and correspond well with the results obtained in the western blotting. CgeA-specific antibodies produced an about 14-kDa band corresponding to native CgeA protein, as well as several bands of higher molecular masses, which most probably represent multimers of CgeA protein since they cannot be visualised in hybridizations with spore coat proteins isolated from the strain BAV08 with inactivation of *cgeA* gene (Figure 4A, lane 2).

### Surface display

**Figure 5:**
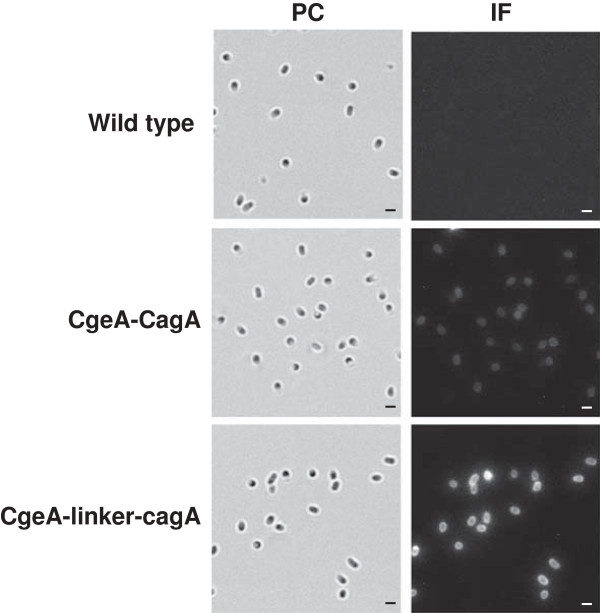
**Localisation of fusion proteins as assessed by immunofluorescence microscopy.** Purified, free spores of wild type strain 168, BAV05 (CgeA-CagA), BAV07 (CgeA-linker-CagA) were visualised by phase contrast (PC) and immunofluorescence (IF) microscopy. The spores were incubated with mouse anti-CagA, followed by anti-mouse IgG-Cy3 conjugates. The same exposure time was used for all IF images. Black and white scale bars – 1 μm.

The surface localization of CgeA-CagA (BAV05) and CgeA-GGGEAAAKGGG-CagA (BAV07) fusion proteins was analyzed by immunofluorescence microscopy of dormant spores of wild type and recombinant strains using anti-CgeA and anti-CagA primary antibodies followed by anti-mouse IgG-Cy3 (Jackson ImmunoResearch Laboratories, Inc). We observed a fluorescent signal around purified dormant spores of both BAV05 and BAV07 strains (Figure 5). These results indicate that both fusion proteins are present on the spore coat surface and are available for antibody binding. It is worth notifying, that fluorescent signal observed in case of BAV07 spores is stronger than in case of BAV05, what clearly suggest, that anchoring of CagA to CgeA protein via helical linker increases the efficiency of surface display of heterologous protein.

## Conclusions

Presented set of vectors is a versatile system for spore surface display of heterologous proteins. Since the successful presentation of proteins on the spore surface depends on its nature, as well as, the properties of obtained fusions it may be necessary to design a panel of plasmids with different spore coat proteins as anchors including or not a peptide linker to enhance the display. A variety vectors included in the presented system facilitates such optimization. It is also important to point out, that all Cot coat proteins used in the system has already successfully been utilized in such applications. Additionally, another coat protein, CgeA, turned out to be useful for presentation of heterologous proteins.

## Methods

### Bacterial strains and transformation

Plasmid amplifications for nucleotide sequencing and subcloning experiments were performed with *Escherichia coli* strains DH5α [25] or BL21 (New England Biolabs, USA). Bacterial strains were transformed by previously described procedures: CaCl_2_-mediated transformation of *E. coli* competent cells [26] and transformation of *B. subtilis*[27]. *Bacillus subtilis* 168 was used for construction of recombinant strains.

### Construction of gene fusions

DNA coding for coat proteins was PCR amplified using the *B. subtilis* 168 chromosome as a template and oligonucleotides listed in Additional file 1: Table S1 as primers. Amplification products were cloned into the vectors listed in the Additional file 1: Table S1. In case of vectors for N-terminal fusions promoter regions of corresponding coat protein genes were separately PCR amplified using oligonucleotides listed in Additional file 1: Table S1 as primers and cloned into appropriate vectors. The DNA coding for *lysA* and *thrC* trophic genes were PCR amplified using appropriate oligonucleotides as primers (Additional file 1: Table S1) and chromosomal DNA of *B. subtilis* strain 168. The *trpC* gene was amplified with using chromosomal DNA of *B. subtilis* 3610 strain with addition of the *p*_*rrnO*_ promoter sequence included in the primers. Obtained PCR fragments harboring a trophic gene along with the promoter were cloned into the proper plasmids (Additional file 1: Table S1). Where necessary the MCS was introduced by cloning a DNA fragment obtained upon annealing of appropriate oligonucleotides (Additional file 1: Table S1). The pCotB vector set was constructed using synthetic gene as described in the additional file. The complete list of final vectors is presented in Table 1.

**Table 1:** List of vectors in the system

**Name**	**Coat gene and type of fusion**	**Integration site and trophic gene**	**Accession number**
pCotB-N	*cotB* N-terminal	*amyE thrC*	[GenBank:KF933395]
pCotB-NL	*cotB* N-terminal + linker		[GenBank:KF933396]
pCotB-C	*cotB* C-terminal		[GenBank:KF933397]
pCotB-CL	*cotB* C-terminal + linker		[GenBank:KF933398]
pCotC-N	*cotC* N-terminal	*lacA trpC*	[GenBank:KF933399]
pCotC-NL	*cotC* N-terminal + linker		[GenBank:KF933400]
pCotC-C	*cotC* C-terminal		[GenBank:KF933401]
pCotC-CL	*cotC* C-terminal + linker		[GenBank:KF933402]
pCotG-N	*cotG* N-terminal	*pyrD lysA*	[GenBank:KF933403]
pCotG-NL	*cotG* N-terminal + linker		[GenBank:KF933404]
pCotG-C	*cotG* C-terminal		[GenBank:KF933405]
pCotG-CL	*cotG* C-terminal + linker		[GenBank:KF933406]
pCotZ-C	*cotZ* C-terminal	*amyE trpC*	[GenBank:KF933407]
pCotZ-CL	*cotZ* C-terminal + linker		[GenBank:KF933408]
pCgeA-C	*cgeA* C-terminal	*amyE trpC*	[GenBank:KF933393]
pCgeA-CL	*cgeA* C-terminal + linker		[GenBank:KF933394]

For construction of *H. pylori* CagA-presenting spores following plasmid was prepared. A 227 bp DNA fragment encoding part of CagA was PCR amplified using *Helicobacter pylori* NCTC 11637 chromosome as a template and oligonucleotides cagA-F and cagA-R (Table 2) as primers. The PCR product was sequentially digested with *BamHI* and *KpnI* and cloned in frame to the 3’ end of the *cgeA* gene carried by plasmid pCgeA-C or pCgeA-CL yielding plasmids pAV05 and pAV03.

**Table 2:** Oligonucleotides list

**Name**	**Sequence (5’-3’)**	**Restriction site**
pRSETAcgeA-F	GTGT**GGATCC**AGCTCTGAAAATGC	*Bam*HI
pRSETAcgeA-R	CGG**GAATTC**TGAAAAGAACGTAACG	*Eco*RI
pLATE52cagA-F	GGTTGGGAATTGCAAGGTATGCTAACGCAAAAAAACCCTG	None
pLATE52cagA-R	GGAGATGGGAAGTCATTATCCTGTAGAAAATGCATTGGC	None
i-cgeA-F	CCC**AAGCTT**ACAGAAGGAGGAGAAAATATG	*Hind*III
i-cgeA-R	CGC**GGATCC**TTTCTACTTTGTCTACATC	*Bam*HI
cagA-F	CGC**GGATCC**GGTATGCTAACGCAAAAAAAC	*Bam*HI
cagA-R	CGG**GGTACC**TTATCCTGTAGAAAATGCATTGGC	*Kpn*I

In bold are the recognition sites for the restriction enzymes indicated in the table. Oligonucleotides used for construction of vectors of the system are listed in Additional file 1: Table S1.

### Chromosomal integration

Appropriate plasmids were linearized by digestion with a single cutting restriction enzyme. Linearized DNA was used to transform competent cells of the *B. subtilis* strain 168. The bacteria were plated on solidified Belitzky minimal medium without tryptophan [28]. Prototrophic clones were the result of a double-crossover recombination event, resulting in the interruption of the non-essential *amyE* gene on the *B. subtilis* chromosome. Several prototrophic clones were tested by PCR using chromosomal DNA as a template and oligonucleotides AmyS and AmyA [29] to prime the reaction. Selected clones were called BAV05 (CgeA-CagA) and BAV07 (CgeA-linker-CagA) and stored for further research.

### Construction of the strain with inactivation of the *cgeA* gene

A 316 bp fragment of the *cgeA* gene was PCR amplified using chromosomal DNA of *B. subtilis* strain 168 as a template and oligonucleotides i-cgeA-F and i-cgeA-R (Table 2) as primes. The obtained PCR product was digested with enzymes *Hind*III and *BamH*I and cloned into the pMUTIN4 vector [30]. The resulting plasmid, pAGW6, was verified by restriction analysis and used for transformation of *B. subtilis* strain 168. Obtained erythromycin-resistant colonies were the result of single crossing-over recombination event leading to the insertion of the plasmid into the chromosome of recipient strain. Selected colonies were PCR-verified for the correctness of the *cgeA* gene inactivation. The resulting strain was called BAV08 and stored for further research.

### Preparation of spores

Sporulation was induced by the exhaustion method in Difco sporulation medium (DSM) as described elsewhere [31]. Sporulating cultures were harvested 24 h after the initiation of sporulation and purified using a lysozyme treatment to break up any residual sporangial cells, followed by washing steps in 1 M NaCl, 1 M KCl and water (twice each), as described by Nicholson & Setlow [31]. PMSF (0.05 M) was included to inhibit proteolysis. After the final suspension in water, spores were treated at 65°C for 1 h to kill any residual cells. The spore suspension was titrated immediately for determination of c.f.u./ml before freezing at -22°C. Using this method, we could reliably produce 6 × 10^10^ spores in 1 liter of DSM culture.

### Extraction of spore coat proteins

Spore coat proteins were extracted from 50 μl of a suspensions of spores at high density (1 × 10^10^ spores per ml) using a decoating extraction buffer as described elsewhere [32]. Extracted proteins were assessed for integrity by SDS-polyacrylamide gel electrophoresis (PAGE) and for concentration by two independent methods: the Pierce BCA Protein Assay (Pierce, USA) and the BioRad DC Protein Assay kit (Bio-Rad, USA).

### Western blotting analyses

Extracted proteins were separated in 12% denaturing polyacrylamide gels, electrotransferred onto a nitrocellulose filter (Roti-NC; ROTH) and used for Western blotting by standard procedures. Western blot filters were visualized by developing with BCIP/NBT according to the manufacturer’s instructions (Thermo Scientific).

### Immunofluorescence microscopy

Samples were fixed directly in the medium as described by Harry et al. [33], with the following modifications: spores were suspended in TE buffer [20 mM Tris/HCl (pH 7.5), 10 mM EDTA] containing 2 mg lysozyme ml. After 3 min of incubation, three washes in PBS (pH 7.4) were performed before blocking with 3% skimmed milk in PBS for 30 min at room temperature and washing another three times in PBS. Samples were incubated overnight at 4°C with mouse anti-CagA antibody, washed three times and then incubated with anti-mouse Cy3-conjugated IgG (Jackson ImmunoResearch Laboratories) overnight at 4°C. After three washes with PBS, samples were loaded on microscope slides previously coated with poly-L-lysine (Sigma). The coverslips were mounted on a microscope slide and viewed using a Zeiss Axioplan fluorescence microscope with the same exposure time for all samples. Images were captured using a camera connected to the microscope, processed with Corel Photo-Paint software and saved in TIFF format.

### Purification of CgeA and CagA and antibody production

The *cgeA* gene of *B. subtilis* was PCR amplified using chromosomal DNA as a template and oligonucleotides pRSETAcgeA-F and pRSETAcgeA-R (Table 2) as primes. The obtained PCR product of 415 bp was digested with enzymes *EcoR*I and *BamH*I and cloned into the commercial vector pRSETA (Invitrogene). The resulting plasmid, pAN17, was verified by restriction analysis and nucleotide sequencing. The protein was purified and used for antibody production following method described previously [21].

The fragment of *cagA* gene of *H. pylori* was PCR amplified using chromosomal DNA as a template and oligonucleotides pLATE52cagA-F and pLATE52cagA-R (Table 2) as primes. The obtained PCR product of 261 bp was cloned into the commercial vector pLATE 52 (Thermo Scientific, Lithuania) using aLICator Ligation Independent Cloning and Expression System (Thermo Scientific). The resulting plasmid, pKH143, was verified by restriction analysis and nucleotide sequencing. The protein was purified and used for antibody production following method described previously [21].

### Ethics statement

This study was carried out in strict accordance with the recommendations in the institutional and national guidelines for animal care and use. The protocol was approved by the Committee on the Ethics of Animal Experiments of the Medical University of Gdańsk (Permit Number: 4/2010). All surgery was performed under isoflurane anesthesia, and all efforts were made to minimize suffering.

## Competing interests

AG, TŁ, MO and KH are authors of Polish patent application no P.403468 filled on 08/04/2013. MS, IP, KH, AI and MO are authors of Polish patent application no P.406271 filled on 26/11/2013, both entitled “Integration vectors, host cell transformed with integration vectors and application of integration vectors and host cell for presentation of fusion proteins on the surface of *Bacillus subtilis* spores”. Patent applications do not alter the authors’ adherence to all the Microbial Cell Factories polices on sharing data and materials.

## Authors’ contributions

AI – participated in study design, constructed pCotG vectors set and wrote the manuscript, IP – constructed pCotB vectors set, MS – constructed pCotC vectors set, AG – constructed pCotZ and pCgeA vectors sets and prepared pCgeA-CagA plasmid, TŁ – constructed pTL01 vector, MO – conceived the study, participated in its design and coordination and helped to draft the manuscript, KH – participated in study design and performed CagA spore surface analysis. All authors read and approved the final manuscript.

## Supplementary Material

Additional file 1: Table S1 Construction of the vectors set. Click here for file
