# Supplementary material for: A system of vectors for Bacillus subtilis spore surface display
Source: Microb Cell Fact. 2014 Feb 24;13:30. doi: 10.1186/1475-2859-13-30 (PMC4015724; doi:10.1186/1475-2859-13-30)
Supplement: Additional file 1: Table S1 — Construction of the vectors set. [file 1475-2859-13-30-S1.pdf]

Additional file 1 Constriction of the vectors set

Table S1 Construction of pCot and pCge vectors

Plasmid	Amplified DNA sequence	Primer name	Primer sequence 5’-3’	Template	Destination
pTL01	Tryptophan biosynthesis pathway gene *trpC* with p_{rrnO} promoter	P2trpC-F	*Eco*RI<br>CCGGAATTCTTTTCAAAAAAGTATTGACCTAGTTAACTAAAAATGTTACTAT TAAGTAGTCGCCAAGGAGGTGAGACCCATGCTTGAAAAAATCATCAAACA AAAGAAAGAAGAAGTGAAAACACTGGTTC	*B. subtilis* 3610 chromosomal DNA	pDG1663 [1]
		P2trpC-R	*Eco*105I<br>CCCTACGTATTACTCCCTAAACAAAGCATGGATTGCTTTACGCTGAGAAGT TTGTCTCATCAATGATTCACCGATAAGTACAGCTCGCGCCCCATGTTCATTG ACAAATGTTAAATGTTCTAAAGAACCGATTC		
**pCotC series**					
pMSW1	Left flanking region of *lacA* gene	N-lacA-up	CGGCCCGATATTTTAGCTG<br>*Hind*III	*B. subtilis* 168 chromosomal DNA	pUC19
		N-lacA-dn	CACTCCCGGAAAGCATGC<br>*Sph*I		
pMSW2	Right flanking region of *lacA* gene	C-lacA-up	TCAGAGCTCCAGCGAGGACACCGTTTC<br>*Sal*I	*B. subtilis* 168 chromosomal DNA	pMSW1
		C-lacA-dn	ATAGAATTCCTGATCCTGCCTCGCTTG<br>*Eco*RI		
pMSW3	Tryptophan biosynthesis pathway gene *trpC*	trpC-up	ACGCTCTAGAAAAAAGTATTGACCTAGTTAAC<br>*Xba*I	pTL01	pMSW2
		trpC-dn	ATACTGCATGCATTACTCCCTAAACAAAGC<br>*Sph*I		
**pCotC-C KF933399**	The promoter and the *cotC* gene	pcotC-up	TACTGAGCTCAGGATAAATCGTTTGGG<br>*Sac*I	*B. subtilis* 168 chromosomal DNA	pMSW3
		cotC-dn	TCAGGGTACCGTAGTGTTTTTTATGCTTTTT<br>*Kpn*I		
**pCotC-CL KF933400**	The promoter and the *cotC* gene	pcotC-up	TACTGAGCTCAGGATAAATCGTTTGGG<br>*Sac*I	*B. subtilis* 168 chromosomal DNA	pMSW3
		cotClink-dn	TCAGGGTACC*AGAACCGCCGCCACC*GTAGTGTTTTTTATGCTTTTT<br>*Kpn*I *linker*		

	with linker at C-terminus				
pMSW4	The promoter and RBS sequence of the *cotC*	pcotC-up	TACTGAGCTCAGGATAAATCGTTTGGG<br>*Sac*I	*B. subtilis* 168 chromosomal DNA	pMSW3
		pcotC-dn	TCAGGGTACCGTAGTGTTTTTATGCTTTTT<br>*Kpn*I		
**pCotC-N KF933401**	The *cotC* gene	cotC-up	TACTGGATCCGGTTATTACAAAAAATACAAAG<br>*Bam*HI	*B. subtilis* 168 chromosomal DNA	pMSW4
		cotCterm-dn	TCAGTCTAGACACAAACAAAAAAGACCC<br>*Xba*I		
**pCotC-NL KF933402**	The *cotC* gene with linker at N-terminus	cotClink-up	TACTGGATCC*GGTGGCGGCGGTTCT*GGTTATTACAAAAAATACAAAG<br>*Bam*HI *linker*	*B. subtilis* 168 chromosomal DNA	pMSW4
		cotCterm-dn	TCAGTCTAGACACAAACAAAAAAGACCC<br>*Xba*I		
**pCotG series**					
pAIW1	Lysine biosynthesis pathway gene *lysA*	lysA-trof-up	TTAAGCATGCGATTTCTTCGATTCTATCTGG<br>*Sph*I	*B. subtilis* 168 chromosomal DNA	pPyr-kan [2]
		lysA-trof-dn	TTAATCTAGATGTGGCAGGTTCTTGTC<br>*Xba*I		
**pCotG-C KF933403**	The promoter and the *cotG* gene	cotG-up	CCCGGATCCCGAGAAAAAATCC<br>*Bam*HI	pKH16	pAIW1
		cotG-dn	CTTGGATCCTTTGTATTTCTTTTTGACTAC<br>*Bam*HI		
**pCotG-CL KF933404**	The promoter and the *cotG* gene with linker at C-terminus	cotGL-up	CCAGGTACCCGAGAAAAAATCC<br>*Kpn*I	pKH36	pAIW1
		cotGL-dn	CCAGAGCTCGTTTGTTGAACTAATGGG<br>*Sac*I		
pAIW2	The promoter and RBS sequence of the *cotG*	promoG-up	ATAGAATTCCAATTTGAAATCC<br>*Eco*RI	*B. subtilis* 168 chromosomal DNA	pAIW1
		promoG-dn	ATAGGATCCCCGAGAAAAATC<br>*Bam*HI		

**pCotG-N KF933405**	The *cotG* gene	cotG2-up	TTAGAATTCTCGAGCTCGGCCACTATTCCCA<br>*Eco*RI	*B. subtilis* 168 chromosomal DNA	pAIW2
		cotG2-dn	TTGGTACCCTATTTGTATTTCTTTTTG<br>*KpnI*		
**pCotG-NL KF933406**	The *cotG* gene with linker at N-terminus	cotG2-lnk-up	AATGAATTCTCGAGCTCGGTGGCGGCGGTTCTCACTATTCCCATTC<br>*Eco*RI	*B. subtilis* 168 chromosomal DNA	pAIW2
		cotG2-dn	TTGGTACCCTATTTGTATTTCTTTTTG<br>*Kpn*I		
**pCotZ series**					
pAGW1	Tryptophan biosynthesis pathway gene *trpC*	trpC-trof-F	GGCGCAATTGTTTCAAAAGTCAATTTGATCAACGG<br>*Mun*I	pTL01	pDL [3]
		trpC-trof-R	ACATGCATGCAAAGTACGTATTACTCCCTAAACAAAGC<br>*Sph*I		
pAGW2	The promoter and the *cotZ* gene	cotZ-F	CGTAGCGAATTCAGTTATCACTCTTGTCCTC<br>*Eco*RI	*B. subtilis* 168 chromosomal DNA	pAGW1
		cotZ-R	GCTTAGGATCCATGATGATGTGTACGATTG<br>*Bam*HI		
pAGW3	The promoter and the *cotZ* gene with linker at C-terminus	cotZ-linker-F	*Eco*RI<br>CCGGAATTCGCAACCCTTATTTCTACAGCAACAAATACACTCGTAGCCATCCTAGTTATCACTCTTGTCCTCTAGGACC	*B. subtilis* 168 chromosomal DNA	pAGW1
		cotZ-linker-R	*Bam*HI *linker*<br>CGCGGATCC*TCCTCCACCTTTCGCTGCTGCTTCTCCTCCACC*ATGATGATGTGTACGATTGATTAATCGAGGATTTAAGC		
**pCotZ-C KF933407**	An insert harboring MCS flanked by sticky ends	MCS-01F	CGGTACCCACGTCAAATCTAGAGTTAACGGTTACCTACGTAAG<br>(*Bam*HI – *Sac*I)	Self-annealed primers	pAGW2
		MCS-01R	GATCCTTACGTAGGTAACCGTTAACTCTAGATTTGACGTGGGTACCGAGCT<br>(*Bam*HI – *Sac*I)		
**pCotZ-CL KF933408**	An insert harboring MCS flanked by sticky ends	MCS-01F	CGGTACCCACGTCAAATCTAGAGTTAACGGTTACCTACGTAAG<br>(*Bam*HI – *Sac*I)	Self-annealed primers	pAGW3
		MCS-01R	GATCCTTACGTAGGTAACCGTTAACTCTAGATTTGACGTGGGTACCGAGCT<br>(*Bam*HI – *Sac*I)		
**pCgeA series**					
pAGW4	The promoter	cgeA-F	CAGCTTAGAATTCTTGAGAGTGAAACATGAG<br>*Eco*RI	*B. subtilis* 168 chromosomal	pAGW1

	and the *cgeA* gene	cgeA-R	CGGGGATCCTGAAAAGAACGTAAC<br>*Bam*HI	DNA	
pAGW5	The promoter and the *cgeA* gene with linker at C-terminus	cgeA-linker-F	*Eco*RI<br>CCGGAATTCAAGCAGAGCCTCTGTCATCATTTAAAAAGCACCCCAGCTTACAACACTTGAGAGTGAAACATGAGATCTCG	*B. subtilis* 168 chromosomal DNA	pAGW1
		cgeA-linker-R	*Bam*HI *linker*<br>CGCGGATCC*TCCTCCACCTTTCGCTGCTGCTTCTCCTCCACC*TGAAAAGAACGTAAACGCTTTCTACTTTGTCTACATC		
**pCgeA-C**<br>**KF933393**	An insert harboring MCS flanked by sticky ends	MCS-02F	AGCTTAGGGCCCATCTAGAAGGTACCGACTCGAGTGTACAGGTAACCAAG (*Bam*HI – *Hind*III)	Self-annealed primers	pAGW4
		MCS-02R	GATCCTTGGTTACCTGTACACTCGAGTCGGTACCTTCTAGATGGGCCCTA (*Bam*HI – *Hind*III)		
**pCgeA-CL**<br>**KF933394**	An insert harboring MCS flanked by sticky ends	MCS-02F	AGCTTAGGGCCCATCTAGAAGGTACCGACTCGAGTGTACAGGTAACCAAG (*Bam*HI – *Hind*III)	Self-annealed primers	pAGW5
		MCS-02R	GATCCTTGGTTACCTGTACACTCGAGTCGGTACCTTCTAGATGGGCCCTA (*Bam*HI – *Hind*III)		

In bold shown names of plasmids composing vector system. GenBank accession numbers are indicated underneath the names.

Construction of pCotB series

A DNA fragment containing *thrC* gene along with the promoter and *cotB* gene flanked by linker-encoding sequences and multicloning sites at both termini was commercially synthesized (Life Technologies, USA) and delivered in a plasmid 12AA35IP_cotB_pMS. *E. coli* DH5α was used for transformation and propagation. The plasmid was digested with *Eco*RV restriction enzyme, cleaned up and subsequently digested with *Sph*I and *Bsi*WI restriction enzymes. Obtained products were separated in agarose gel and appropriate fragment was isolated from the gel. The fragment was cloned into *Sph*I/*Bsi*WI-digested pDL vector resulting in pIW plasmid.

pCotB-NL

pIW plasmid was digested with *Eco*RI and *Xho*I, the sticky ends of the obtained product were filled in with Klenow fragment and re-ligated.

pCotB-N

pCotB-NL plasmid was digested with *Kpn*I to remove linker-encoding sequence and re-ligated.

pCotB-CL

pIW plasmid was digested with *Kpn*I, the sticky ends of the obtained product were filled in with Klenow fragment and re-ligated.

pCotB-C

pCotB-CL plasmid was digested with *Eco*RI to remove linker-encoding sequence and re-ligated.

References

1. Guérout-Fleury AM, Frandsen N, Stragier P: **Plasmids for ectopic integration in *Bacillus subtilis*.** *Gene* 1996, **180**:57-61.
2. Middleton R, Hofmeister A: **New shuttle vectors for ectopic insertion of genes into *Bacillus subtilis*.** *Plasmid* 2004, **51**:238-245.
3. Yuan G, Wong SL: **Regulation of *groE* expression in *Bacillus subtilis*: the involvement of the sigma A-like promoter and the roles of the inverted repeat sequence (CIRCE).** *J Bacteriol* 1995, **177**:5427-5433.
